# Conducting complex intervention trials in populations at risk of diminished capacity

**DOI:** 10.1186/1745-6215-16-S2-P93

**Published:** 2015-11-16

**Authors:** Amanda Farrin, Suzanne Hartley, Liz Graham, Rachael Kelley, Claire Surr, Alys Griffiths, Louise Bryant

**Affiliations:** 1 University of Leeds, Leeds, UK; 2 Leeds Beckett University, Leeds, UK

## 

Implementing clinical trials involving participants with diminished capacity, such as elderly residents in care homes, stroke survivors, those with a learning disability, can be challenging in terms of balancing complex service and patient needs with methodological rigor. The key challenges identified here are the appropriate tailoring of recruitment approaches, data collection and participant risk monitoring.

Optimising the consent process for those with varying levels of comprehension and communication is complex. For example, conducting research with participants with dementia requires special consideration around the salient points for inclusion in information sheets (including multiple versions to cater for varying levels of capacity) and utilising different formats to aid communications. Consideration also needs to be given to optimising provision of information to consultees where capacity is lacking.

Data collection strategies need to be adapted to ensure understanding, to utilise appropriate data sources and maximise data return. Participants may have limited ability to provide self-report data, and the way in which they are able to respond will vary. Simplified tools, visual prompt aids, or the collection of proxy data should all be considered. There is also the need to be vigilant for other issues arising from research with this population, such as safe guarding concerns, which will require specialised researcher training and clear onward reporting processes.

We will describe and discuss how trials involving populations at risk of diminished capacity have adapted their recruitment strategies, data collection approaches, and linked with service providers to ensure participant safety.

